# Examining Preschoolers’ Emotion Regulation Strategies: Psychometric Properties of the Translated Dutch Early Emotion Regulation Behavior Questionnaire (EERBQ-Dutch)

**DOI:** 10.3390/children12040494

**Published:** 2025-04-11

**Authors:** Iris Heselmans, Marie Van Gaever, Hana Hoogers, Kurt Eggers

**Affiliations:** 1 Stuttering Research Group, Department of Rehabilitation Sciences, Ghent University, 9000 Ghent, Belgium; marie.vangaever@ugent.be (M.V.G.); hana.hoogers@ugent.be (H.H.); kurt.eggers@ugent.be (K.E.); 2 Department of Speech-Language Pathology, Thomas More University College, 2000 Antwerp, Belgium

**Keywords:** emotional regulation, emotional reactivity, preschoolers, early emotion regulation behavior questionnaire

## Abstract

Objectives: Early difficulties in emotion regulation are associated with psychopathological, broader social, and developmental outcomes, underscoring the need for robust assessment tools at a young age. However, most of the existing instruments for preschoolers measure emotion regulation in general, without focusing on specific emotion regulation strategies. This study addresses a critical gap by validating a Dutch version of the Early Emotion Regulation Behavior Questionnaire (EERBQ), enabling researchers and practitioners to assess preschoolers’ emotion regulation strategies in both positive- as well as negative-emotion-eliciting situations outside of laboratory settings. Methods: Through a rigorous back-translation process, the parental questionnaire was adapted into Dutch (EERBQ-Dutch) and subsequently validated with a sample of 299 Dutch-speaking caregivers of typically developing 2–7-year-old children. The test underwent psychometric analysis including inter-item correlations, item–total correlations, test–retest reliability, and confirmatory factor analysis. Finally, potential sociodemographic predictors (i.e., age, sex, and socioeconomic status (SES)) of specific emotion regulation strategies were investigated. Results: Psychometric analyses demonstrated strong reliability and validity, and a factor structure consistent with the original English questionnaire. Age and sex were found to be significant predictors of certain emotion regulation strategies, with more proficient use of adaptive emotion regulation strategies over time and girls employing more Verbal Help-Seeking and less Physical Venting and Reactivity compared to boys. SES only contributed to Emotional Reactivity with a higher SES predicting more Reactivity. Conclusions: Our findings support the EERBQ-Dutch as a reliable and culturally appropriate instrument for assessing early emotion regulation and provide insight into key predictors of emotion regulation strategies.

## 1. Introduction

Emotion regulation (ER) has been a subject of research for over three decades [1,2,3,4,5,6] and has been studied across many (sub-)disciplines [7,8]. Due to the complexity of this construct, it encompasses multiple definitions [1,6]. The umbrella term refers to the processes involved in the initiation, maintenance, and modulation of emotional responsiveness, both positive and negative [9,10]. Hence, strong ER allows a person to not just surrender to emotions but to be able to influence them. ER not only influences the intensity, duration, and/or quality of the emotions themselves [6,11], but also affects other psychological processes, such as social interaction and memory [12]. Although some argued that distinguishing the construct of ER from the construct of emotion is challenging [13,14], Cole et al. [12] contended that a distinction is possible, as emotions exert different effects depending on how they are regulated. Encountering a bear is a simple example illustrating the difference between both constructs: the emotion of fear triggers a physiological response, activating the sympathetic nervous system to prepare the body for running, whereas ER may lead to choosing not to flee.

From a theoretical perspective, ER has been integrated into various (bio)psychological models, including Rothbart’s temperament model [15]. Rothbart [16] defined temperament as “the constitutionally based differences in reactivity and self-regulation” (p. 10). The term ‘constitutional’ refers to the biological bases of temperament. Within this model, ‘reactivity’ denotes motor, emotional, and attentional responses to both internal and external stimuli and is further divided into positive reactivity and negative reactivity, reflecting a positive or negative sensitivity to environmental factors. Self-regulation, which overlaps with ER, refers to the processes that facilitate or inhibit reactivity [2,17,18], such as response inhibition, attentional shifting, and executive control [19]. In essence, reactivity describes the extent to which a child responds to an emotion-evoking stimulus, while ER pertains to the degree to which the child can modulate this response, upregulating positive emotions (e.g., fostering joy or optimism) or downregulating negative emotions (e.g., reducing anxiety or frustration).

### 1.1. Emotion Regulation in Early Childhood

Although preschoolers might not be the first image that comes to mind when thinking of a proficient ER user, young children already display a wide range of ER strategies [9,20,21,22,23,24,25,26]. The preschool years are critical for ER development, as children become more aware of emotions and their connections to external behavior and psychological states [27,28,29,30,31,32]. During this time, growth in executive functioning skills, such as regulating attention, inhibiting behavior, and retaining information in the working memory, provide additional underpinnings for certain ER strategies [33]. However, young children mainly use external behavioral strategies [11,34], which require less cognitive effort [9]. Although effective in reducing emotional arousal, they can be socially inappropriate. For example, “physical venting” [35], such as running, jumping, or kicking, is commonly used by preschoolers in response to anxiety or frustration, which reduces emotional intensity but might not be socially appreciated. Thus, expanding our understanding of ER strategy use during the early developmental years is crucial. As children progress into middle childhood and especially adolescence, they develop more sophisticated and internalized ER strategies [11,36,37,38]. Although the general consensus seems to support that ER abilities improve with age [39], there are conflicting findings with some studies not reporting age effects [40].

Apart from age and maturing processes, sex differences also influence the use of ER strategies, with preschool boys favoring behavioral strategies, undertaking action to manage the emotion, while girls prefer social support strategies, that is, seeking intervention of another person to manage the emotion [41]. Also, among school-age children, girls, compared to boys, employ more ER facing sadness, fear, and anger, though this pattern reverses during adolescence [36]. These differences have been linked to genetic (e.g., hormonal factors) and aging effects [42], the influence of education on gender role learning [43,44], as well as societal expectations in shaping the behaviors of men and women in various contexts [45]. While most studies seem to support that females tend to use more emotion-focused coping strategies (e.g., rumination, emotional expression, and emotional support seeking) and males may engage more in problem-solving and avoidance strategies [46,47], aligning with gender socialization theories [43,44,45], the literature is not unequivocal, with studies finding conflicting sex-related findings for specific ER strategies [7,36,40,47,48,49,50,51]. This triggered debate about whether these differences are more about socialization than biology. In addition, Nolen-Hoeksema [46] argued that when studies do find sex differences in ER, they are small even when they are statistically significant, and discussed how common assumptions about sex-differences in the general literature should be considered with caution. Therefore, further research is needed to bridge gaps in our current understanding.

The meta-analysis of Matthews et al. [40] identified 18 contributing factors to ER employment. Even in preschoolers, beyond age and sex, several additional factors influencing the usage of ER (strategies) have been identified, such as motor, language (verbal and non-verbal), emotional, and cognitive developmental factors, as well as family factors and the socioeconomic status (SES) [12,41,52,53,54,55,56,57,58]. More advanced gross motor skills, for example, correlate with greater ER skills, mediated by executive function [54]. Non-verbal intelligence seems to facilitate the use of advanced ER strategies, such as cognitive reappraisal [41]. High receptive vocabulary skills have been associated with different ER strategies depending on children’s age, namely, increased use of social support strategies in 3- and 4-year-olds and a shift towards behavioral strategies which require more advanced linguistic and cognitive skills in 5- and 6-year-olds. Lastly, a higher SES correlates to a higher usage of adaptive ER strategies and a lower employment of maladaptive ER strategies [56]. Apart from the fact that this list of influencing factors is not exhaustive, not all studies are unequivocal on the specific role of each of the factors. For example, although SES seems to play a role in the ER strategy use [52,56], there are conflicting findings and studies suggested that SES might only contribute during certain periods in life [59]. Therefore, there is still a need for research within this domain.

### 1.2. Emotion Regulation and (Psycho)pathology

Although children develop greater ER skills over time, not all children develop the ability to regulate emotions effectively [60]. Challenges in ER are often associated with psychopathology, developmental disorders, and health issues [1,61,62,63,64,65,66,67], including internalizing [68] and externalizing problems [69,70], even in young children and adolescents [71,72,73,74]. The literature on ER and (psycho)pathology often distinguishes between adaptive and maladaptive strategies [38,48,75,76]), referring to the effectiveness of strategies in achieving the desired emotional, behavioral, or cognitive outcome [1,76,77]. Aldao and colleagues [75,78,79,80] provide examples of adaptive ER strategies, such as acceptance (acknowledging the emotion without trying to change it), cognitive reappraisal (reframing a situation to change its emotional impact), and problem-solving behavior (conscious, focused attempts to change a situation). Examples of maladaptive ER strategies described by the same authors are suppression (inhibiting the emotional experience and expression), avoidance (trying to escape or avoid unwanted situations/experiences), nonacceptance (experiencing secondary negative emotions as a result of an emotional experience), and anxiety and rumination (unproductive, repetitive thoughts).

The pervasive reliance on maladaptive ER strategies has been associated with symptoms of anxiety [66,67,73,74,75,81,82], depression [48,66,71,72,74,81,83,84,85], and behavioral problems [34,60,74,83,86], as well as psychiatric disorders [64,87] such as generalized anxiety disorder [88,89], depressive disorder [90,91], borderline personality disorder [92,93,94], bipolar disorder [95,96], and eating disorder [97,98,99,100,101]. In addition, different developmental disorders have been associated with ER difficulties. For example, ADHD has been linked to extremely low levels of ER [88,102] and the maladaptive strategy of giving up (doing nothing more and thinking there is nothing you can do to change your anger/anxiety/grief). Developmental stuttering, as another example, has also been linked to lower levels of ER, with, for example, lower levels of frustration tolerance [103].

Given that early emotional development plays a key role in later social, emotional, and cognitive outcomes, access to a reliable and valid instrument to cost-effectively monitor ER strategies in very young children could significantly contribute to the existing literature and be valuable to possibly enhance targeted prevention and intervention efforts in the future. This perspective is supported by research highlighting that ER is an important process that should be considered when examining the onset and persistence of developmental and psychiatric disorders as well as its treatment [65,104,105,106,107].

### 1.3. Measuring Emotion Regulation in Early Childhood

ER in preschool children can be assessed through parental questionnaires, direct observation, and psychophysiological measures [35,108,109,110]. Although psychophysiological measures show promise [108,111,112,113,114,115], the application seems challenging with younger children and remains inaccessible in clinical practice. Consequently, a vast amount of early childhood studies relies on observational experimental methods, given young children’s limited capacity for self-reflection and reporting of emotional experiences [6,10,116]. However, distinguishing between ER and emotional valence during observation poses a significant challenge [12]. Furthermore, many of these studies are conducted in controlled, artificial settings designed to elicit strong emotions, which not only limits their applicability in clinical contexts but also may inflate the likelihood of detecting significant results not resembling the ER levels in the home environment.

Parental questionnaires offer a more practical alternative and are widely used in both research and clinical settings. There is a wide range of parental questionnaires investigating ER in (early) childhood, as summarized by Zentner and Bates [117] and Adrian et al. [116]. For preschoolers specifically, for example, there are the Children’s Behavior Questionnaire (CBQ) [18] and the Behavioral Inhibition Questionnaire [118]. Apart from the fact that some of these questionnaires are rather dated, few of them have been translated into Dutch (e.g., CBQ). In addition, these questionnaires do not specifically assess ER, let alone distinguish between particular ER strategies.

A widely used caregiver questionnaire that does specifically focus on ER is the Emotion Regulation Checklist [119]. However, this questionnaire is not available in Dutch and primarily offers general insights into children’s regulation abilities without addressing the use of specific ER strategies in distinct emotional contexts. This distinction is crucial, as research indicates that different strategies are employed depending on the emotion elicited [39,77]. Furthermore, most of the questionnaires that assess specific ER strategies in emotion-eliciting situations, for example the Children’s Emotional Management Scales [74,120,121], focus solely on negative emotions, which might be a shortcoming to our understanding of daily ER employment. To address these gaps, Perry and Dollar [35] developed the Early Emotion Regulation Behavior Questionnaire (EERBQ), designed for caregivers of preschoolers aged 2 to 6 years, assessing 8 ER strategies across 12 emotion-eliciting scenarios. These eight strategies encompass adaptive and maladaptive strategies that are questioned in relation to both positive as well as negative emotions. This is particularly valuable, as to date it is not exactly clear if the same ER strategies are used in both positive- and negative-emotion-eliciting situations, given that the majority of studies predominantly focus on negative emotions and their regulation [122]. Apart from assessing eight ER strategies, the EERBQ provides six additional questions to attain an overall Emotional Reactivity score.

Considering the lack of standardized Dutch parental-report tools for ER strategy assessment related to both positive and negative emotions in preschoolers, the EERBQ was translated. Since studies show that the usage of ER strategies differs across various cultures [123,124,125,126], there was a need to validate the translated questionnaire and investigate its psychometric strengths. Furthermore, given the inconsistent findings regarding age, sex, and SES as potential contributors to ER strategy use, investigating these demographic factors remained highly relevant.

To address these gaps in our understanding of how young children regulate their emotions in both positive- as well as negative-emotion-eliciting situations, to determine what the influence is of sociodemographic aspects, as well as to overcome the scarcity of Dutch questionnaires assessing both adaptive and maladaptive strategies in preschoolers, the EERBQ was translated into Dutch, resulting in the Dutch Early Emotion Regulation Behavior Questionnaire (EERBQ-Dutch).

The present study aims to (1) evaluate the psychometric properties of the EERBQ-Dutch, including test–retest reliability, and (2) explore potential correlations between ER strategies and demographic variables, i.e., age, sex, and SES.

## 2. Methods

### 2.1. Participants

A total of 299 Dutch-speaking parents (96% mothers, 2% fathers, and 2% parent pairs) of typically developing preschoolers between the ages of 2 and 6; 11 years old (48% boys and 52% girls; *M* = 53 months; *SD* = 14) completed the EERBQ-Dutch. When parents reported any developmental, speech, language, and/or hearing disorder in their preschooler, they were excluded. Through a demographic questionnaire, the inclusion criteria were guaranteed. All classes of SES, as well as marital status, were reached. For measuring test–retest reliability, 64 parents completed the questionnaire for a second time between four to eight weeks after the first test-taking. It was ensured that the same parent as on the first test-taking completed the questionnaire.

### 2.2. EERBQ Translation

The EERBQ-Dutch was developed through the process of back-translation. Initially, the questionnaire was translated from English into Dutch by a Dutch-speaking (Flemish) researcher with a CEFR proficiency level C1. This Dutch version was translated back into English by a second Dutch-speaking (Flemish) researcher with an English CEFR proficiency level C2. The original English version of the EERBQ was then compared with the back-translated English version to identify possible discrepancies. Apart from the rigorous back-to-back translation process, to ensure cultural appropriateness of the EERBQ-Dutch, each hypothetical situation and each item was discussed within the research team to ensure that the constructs being measured in the original survey were conceptually equivalent and culturally relevant for both Flanders (Dutch-speaking part of Belgium) and the Netherlands. All situations were found to be applicable for preschoolers in these countries and no significant discrepancies between items were found, aligning with the existing literature suggesting high cultural and psychological homogeneity between the USA and Western European countries (including Belgium and the Netherlands), as both nations are part of the WEIRD societies (Western, Educated, Industrialized, Rich, and Democratic) [127,128,129].

The minor linguistic differences that were observed were traced to nuances in the Dutch language. These discrepancies were discussed within the research team, with the most appropriate linguistic choices for the Dutch language area being selected in each case. Once the Dutch version was finalized, it underwent a review process involving six Dutch-speaking individuals, three from the Netherlands and three from Flanders, representing a diverse age range (22–56 years), ensuring the translation was properly understood and usable in both Dutch-speaking countries. Feedback from this group led to additional minor revisions to better align the questionnaire with typical language usage in the Netherlands. The result of this process was the finalized EERBQ-Dutch.

### 2.3. Structure of EERBQ-Dutch

The EERBQ-Dutch, in accordance with the original EERBQ [35], measures the consistent usage of ER strategies of preschoolers outside of a clinical setting in 12 hypothetical everyday situations. These scenarios were determined based on a parental survey questioning typical scenarios eliciting strong negative and positive emotions. Based on conceptual definitions of ER strategies routinely observed in clinical settings and the structure of widely used ER measures, for example the Coping with Children’s Negative Emotion Scale [130], eight ER strategies were selected (see Table 1). These eight strategies are questioned in both positive- and negative-emotion-eliciting situations, more specifically, anger, sadness, fear, and excitement. In short, each of the 8 ER strategies is questioned under each of the 12 hypothetical scenarios and is scored by the parent with the probability with which the child would react with the 8 ER strategies on a 1 (very unlikely) to 7 (very likely) Likert scale.

Each of the eight strategies is assigned an overall score, calculated by summing the individual scores of each item questioning the strategy, ranging from 12–84. The higher the total score, the more likely a child will use this strategy in an emotion-eliciting situation.

In addition to ER strategies, the questionnaire includes an Emotional Reactivity scale consisting of 6 specific questions rated on a 7-point Likert scale (1 = strongly disagree, 7 = strongly agree). A total Emotional Reactivity score is calculated ranging from 6 to 42, with higher scores indicating greater reactivity. Three out of the six Emotional Reactivity questions are reverse-scored.

### 2.4. Procedures

Envelopes encompassing an informed consent form, a demographic questionnaire, and the EERBQ-Dutch were distributed in preschools in Flanders. Participants signed an information and consent form, completed a demographic questionnaire, and the EERBQ-Dutch. After finalizing all documents, parents handed the envelope to the preschool teacher and the researcher picked them up.

### 2.5. Data Analyses

To examine the psychometric properties of the EERBQ-Dutch, descriptive statistics and internal reliability measures, more specifically, Cronbach’s alpha (α) and item–total correlations, were computed for each strategy as well as for the Emotional Reactivity scale. Furthermore, test–retest reliability was determined using the interclass correlation coefficient (ICC) with a two-way mixed-effects analysis [131], based on the average scores for each ER strategy and for the Emotional Reactivity scale over the two testing moments. Missing data were handled by mean imputation, since missing values did not exceed 2% per item.

A confirmatory factor analysis (CFA) was performed using IBM statistics AMOS (version 29) with the Maximum Likelihood (ML) estimation method. Missing data did not exceed 3% for each item and were therefore handled via Full Information Maximum Likelihood. To enhance comparability with the original English EERBQ, a similar analysis to that of the original EERBQ-study [35] was performed: separate CFAs were conducted on the covariance matrices of the 12 raw item scores per behavioral strategy. Because each strategy is questioned under the same hypothetical situations, the structure of the questionnaire suggests high correlations between behavioral strategies, making a fully inclusive CFA model less suitable. Additionally, composite reliability scores (CR) were analyzed to further investigate the internal consistency for each latent construct in this CFA. This measure is particularly suitable to provide an accurate estimate when factor loadings are unequal, since it does not assume that all items contribute equally to the construct [132,133,134,135].

Lastly, to determine whether age, sex, and SES significantly predict the use of certain ER strategies, multiple linear regressions were conducted. SES was determined based on the Hollingshead Four Factor Index of Social Status [136], taking into account the family constitution, educational level, and the occupational status of the household.

## 3. Results

### 3.1. Internal Reliability

Descriptive statistics (Table 2) showed that Physical Help-Seeking (*M* = 5.14, *SD* = 0.95), Mindfulness (*M* = 4.76, *SD* = 1.37), and Verbal Help-Seeking (*M* = 4.73, *SD* = 1.34) strategies had the highest mean scores, whereas Self-Soothing (*M* = 2.08, *SD* = 1.31) and Avoidance (*M* = 2.15, *SD* = 0.87) had the lowest. Cronbach’s alpha showed acceptable to excellent internal reliability (0.79–0.94), with Avoidance having acceptable reliability (*α* = 0.79) and all other strategies good to excellent consistency. The Emotional Reactivity scale showed moderate reliability (0.66). Overall, results suggest that the subscales exhibit strong internal consistency, supporting their reliability for measuring the intended ER strategy.

### 3.2. Item–Total Correlation

The Self-Soothing subscale demonstrated the strongest item–total correlations (see Table 3), ranging from 0.63 to 0.79, indicating that all items contributed meaningfully to the overall construct. Similarly, Mindfulness (0.49 to 0.77), Distraction (0.34 to 0.60), Verbal Help-Seeking (0.50 to 0.74), Physical Help-Seeking (0.33 to 0.58), and Verbal Venting (0.43–0.74) showed correlations exceeding the 0.30 threshold [137,138], further supporting their internal reliability. Both Physical Venting (0.25 to 0.69) and Avoidance (0.23 to 0.63) showed more variance, with not all items exceeding the 0.30 threshold but still above the 0.15 threshold [139], suggesting potential room for refinement. Because of the fixed structure of the questionnaire, no items were excluded. Overall, these findings support internal consistency.

With regards to the Emotional Reactivity scale, most items demonstrated acceptable correlations, exceeding the 0.15 threshold. Questions 5 and 6 showed notably lower correlations (0.18 and 0.13, respectively), suggesting that these items may not align as well with the overall construct measured by the scale.

### 3.3. Test–Retest Reliability

Table 4 shows that the interclass correlation coefficient (ICC) values ranged from 0.760 to 0.938, with the measure for Verbal Help-Seeking demonstrating the highest stability (ICC = 0.938), whereas the Avoidance strategy showed the lowest reliability (ICC = 0.760). The remaining strategies all exhibited good to excellent test–retest reliability. Lastly, all ICC values were statistically significant (*p* < 0.001), indicating that the observed reliability estimates are unlikely to be due to chance. Similarly, the Emotional Reactivity scale showed strong test–retest reliability (ICC = 0.855; *p* < 0.001).

Overall, these results suggest that the measures for each ER strategy, as well as the Emotional Reactivity scale, possess good to excellent test–retest reliability, with one strategy scoring acceptable reliability. The consistently high ICC values, coupled with narrow confidence intervals, support the use of this instrument.

### 3.4. EERBQ-Dutch Factor Structure

**Figure 1:**
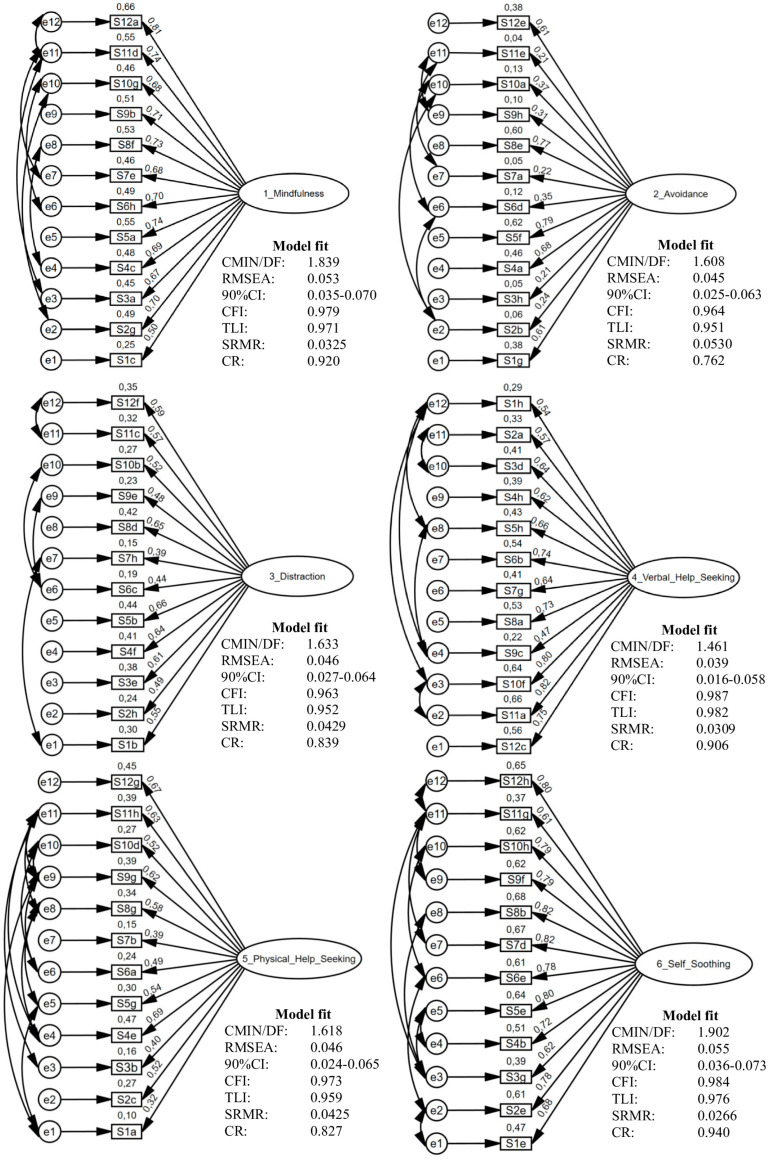

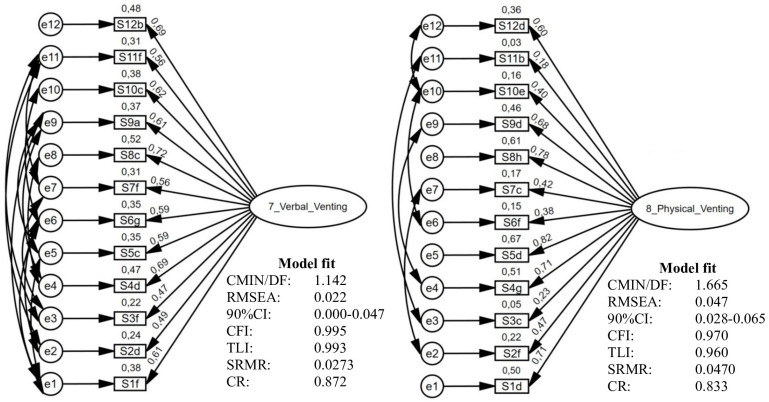
CFA models with error covariances (bidirectional arrows), model fit indices, composite reliability scores, and standardized factor loadings and error variances.

To examine the factorial structure of the EERBQ-Dutch, a confirmatory factor analysis (CFA) was conducted using maximum likelihood estimation in AMOS. Model fit was evaluated based on multiple fit indices, including the Chi-Square Minimum Discrepancy divided by Degrees of Freedom (CMIN/DF), Comparative Fit Index (CFI), Tucker–Lewis Index (TLI), Root Mean Square Error of Approximation (RMSEA) with 90% confidence intervals, and Standardized Root Mean Square Residual (SRMR), as visualized in Figure 1. Model fit was optimized by introducing error covariances between items of the same strategy based on modification indices, visualized with bidirectional covariance arrows. All error covariances were theoretically justified. Apart from the fact that correlations between items were expected because of the structure of the questionnaire, most error covariances occurred between items that question the same strategy in a hypothetical situation eliciting the same emotion. For example, with regards to Verbal Venting, a correlation (0.25) was found between two items questioned in a hypothetical situation eliciting anger, namely, ‘If my child becomes angry because he/she is hurt and can’t go outside to play with siblings/friends’ and ‘If my child is angry because he/she wants a toy/snack at the store and I will not buy it’.

All strategies demonstrated generally acceptable to excellent model fit. The CMIN/DF ranged from 1.142 to 1.902, indicating good model fit across strategies. RMSEA values (0.022–0.047) showed good fit for all strategies, apart from Mindfulness (0.053) and Self-Soothing (0.055) having acceptable fit. The CFI and TLI were consistently high, with values ranging respectively from 0.963 to 0.995 and 0.951 to 0.993, suggesting strong model performance. Lastly, SRMR values remained below 0.0530, indicating minimal residual error.

Factor loadings varied across strategies, with most standardized estimates falling within acceptable to excellent ranges, exceeding the recommended threshold of 0.40 [133]. Strategies such as Mindfulness, Self-Soothing, and Verbal Help-Seeking demonstrated strong factor loadings (λ = 0.50–0.82), while for Avoidance (λ = 0.21–0.79) and Physical Venting (λ = 0.18–0.82) some items exhibited lower factor loadings. Due to the fixed structure of the questionnaire, all items were retained in the model. Despite variations, the overall pattern of results supports the factorial validity of the translated questionnaire. Lastly, CR scores are presented in Figure 1. All CR values exceeded the 0.70 threshold [140], showing strong internal consistency for each latent construct in this CFA. In summary, the CFA results provide evidence for the distinct factor structures of the eight ER strategies of the EERBQ-Dutch, suggesting that the questionnaire adequately captures distinct ER strategies.

### 3.5. Relation Between EERBQ and Demographic Variables

Multiple regression analyses were conducted to examine whether age, sex, and SES predict different ER strategies (Table 5). Regression models were significant for Mindfulness (*F*(3, 275) = 16.793, *p* < 0.001), Verbal Help-Seeking (*F*(3, 276) = 30.464, *p* < 0.001), Verbal Venting (*F*(3, 276) = 8.681, *p* < 0.001), Physical Venting (*F*(3, 276) = 7.495, *p* < 0.001), and Emotional Reactivity (*F*(3, 278) = 3.060, *p* = 0.017). Demographics explained the highest variance for Verbal Help-Seeking (R^2^ = 0.249) and Mindfulness (R^2^ = 0.155), and the lowest for Emotional Reactivity (R^2^ = 0.036) and Verbal Venting (R^2^ = 0.064), with Physical Venting somewhere in the middle (R^2^ = 0.106).

For Mindfulness and Verbal Venting, age was the only significant predictor (respectively, β = 0.371, *p* < 0.001; β = −0.018, *p* < 0.001), with older participants being more likely to engage in Mindfulness and less in Verbal Venting. Age and sex were significant predictors for Verbal Help-Seeking (respectively, β = 0.043, *p* < 0.001; β = 0.297, *p* = 0.035) and Physical Venting (respectively, β = −0.012, *p* < 0.001; β = −0.427, *p* < 0.001), with sex as strongest predictor. Girls use more Verbal Help-Seeking than boys but engage less in Physical Venting; older participants use more Verbal Help-Seeking and less Physical Venting.

SES (β = 0.012, *p* = 0.037) and sex (β = −0.282, *p* = 0.013) were significant predictors of Emotional Reactivity, indicating that boys and children with a higher SES are more emotionally reactive. No significant models were found for Avoidance, Distraction, Physical Help-Seeking, or Self-Soothing (*p* > 0.050), indicating that age, sex, and SES did not significantly predict these strategies.

Overall, these findings suggest that age and sex are key predictors of certain ER strategies, with older individuals showing more adaptive strategies, such as Mindfulness, and less reliance on Venting, while sex differences emerge particularly in Help-Seeking and Emotional Reactivity. SES appears to play a more limited role.

## 4. Discussion

The aim of the present study was to evaluate psychometric properties, including test–retest reliability, of the Dutch version of the EERBQ, to compare the factor structure of the EERBQ-Dutch to the original EERBQ [35], and to investigate possible demographic predictors of ER strategies and Emotional Reactivity.

### 4.1. Psychometric Properties

Internal reliability analysis showed a good to excellent consistency for all strategies, with Avoidance and Emotional Reactivity scoring somewhat lower, pointing to acceptable reliability. Overall, this suggests that the items reliably measure their intended ER strategy. These internal consistency scores align with those reported in the original study [35]. Specifically, when categorized by reliability levels (e.g., acceptable to excellent), each strategy’s internal consistency in our study falls within the same category as in the original study.

Item–total correlations were all within an acceptable range, with the strongest correlations for Self-Soothing, while one item of the Emotional Reactivity scale did not reach the 0.15 threshold [139]. Generally, these results indicate that each item contributed meaningfully to the overall ER strategy. Since the original study [35] did not examine inter-item correlations, the inclusion of this analysis provides additional power to the questionnaire’s reliability.

Another important contribution of our study is the assessment of test–retest reliability. ICC analysis indicated strong to excellent stability of the ER strategies and the Emotional Reactivity scale over time. This finding strengthens the validity of the EERBQ-Dutch and provides additional evidence for the applicability in both future research as well as clinical settings.

### 4.2. Confirmatory Factor Analysis

CFA was conducted to compare the factor structure of the EERBQ-Dutch to the original EERBQ [35]. Fit indices showed perfect model fit for all eight strategies. Although not all factor loadings exceeded the commonly expected threshold of 0.40 [133], most items represented their intended strategy well. For the Avoidance strategy in particular, multiple items did not exceed the 0.40 threshold but still exceeded the 0.15 threshold, as mentioned by Clark and Watson [139]. This aligns with the original EERBQ-study, which also found lower factor loadings for the same items within the Avoidance scale. Therefore, this issue may not be unique to a particular context but might point to potential model issues with item redundancy or the possibility that some items may have been too broad to assess the Avoidance strategy accurately. From a more theoretical perspective, these results might confirm studies pointing to psychosocial similarities between the US and Belgium [127,128,129], where avoidance might be seen as a less adaptive and socially accepted strategy, leading to less variance in responses to those items. Future studies could take this into account and explore possible refinements of the Avoidance strategy. Despite the weaker factor loadings for some items of certain strategies, the collective contribution of all items for each strategy still ensured perfect overall reliability of the construct, as shown by the excellent composite reliability scores.

Our CFA findings largely replicate the original study [35] and suggest that the factor structure remains consistent across samples. However, the original study reported poor model fit and weak factor loadings for Self-Soothing, whereas our results indicate excellent model fit and strong standardized factor loadings for this strategy. This discrepancy may be due to improved item clarity in the translation process. Additionally, subtle cultural differences in the understanding or use of Self-Soothing may have contributed to the improved factor structure as coping mechanisms can vary across populations [124,125]. Future studies could investigate whether these differences stem from linguistic nuances, cultural variations in coping behaviors, or more specific sample characteristics. Overall, our findings confirm the structure of the translated questionnaire and its stability across studies and languages. This reinforces the reliability of the questionnaire in a new context.

### 4.3. Demographic Predictors for ER Strategies

Our aim was to determine possible demographic predictors for ER and compare these results to the original EERBQ-study [35]. Before delving into the specifics of the predictors investigated in this study (i.e., age, sex, and SES), it is noteworthy to mention that the mean scores we observed for each of the eight ER strategies, as well as the overall Emotional Reactivity scale, were largely consistent with those of the US sample from the original EERBQ-study [35]. More specifically, the largest mean difference of 0.58 was found for the Distraction strategy and six of the eight strategies did not exceed a mean difference of 0.22, suggesting that the patterns of general ER strategy use we found are highly similar to those observed in the original sample. This aligns with the existing literature pointing to high psychological and cultural similarities between WEIRD societies [127,128,129]. However, while these results indicate high consistency between the two samples, this similarity does not necessarily imply the absence of intercultural variability. More nuanced differences might emerge when specifically encountering cultural factors by adding additional variables, such as parenting style, coregulation, and cultural norms. Furthermore, these similarities should be interpreted with caution, as the generalizability of mean scores may be limited since prior studies, for example, suggested ER strategy differences between Western and East Asian cultures [126].

Age significantly predicted the use of Mindfulness, Verbal Help-Seeking, and Verbal and Physical Venting. Older participants were more likely to engage in Mindfulness and Verbal Help-Seeking and less in Verbal and Physical Venting. These results align with both the original EERBQ-study [35], as well as the general literature indicating that children become more proficient in the use of adaptive, more sophisticated strategies over time [141]. However, this trend seems to shift during adolescence, when more complex but maladaptive strategies, like self-blame, tend to emerge [36]. This pattern then transitions back to a more adaptive strategy use in adulthood [142]. Some studies have reported additional age effects we did not observe, such as a decline in Self-Soothing during early childhood [25]. This discrepancy may be due to possible differences in sample characteristics, cultural influences, or measurement methods. For example, cultural norms could influence how children engage in Self-Soothing, as studies showed that some cultures emphasize external support through interpersonal ER [143]. Furthermore, certain ER strategies may be more prominent in the group we examined, potentially overshadowing the role of Self-Soothing.

In addition, sex was a significant contributor to Verbal Help-Seeking, Physical Venting, and Emotional Reactivity, with girls using more Verbal Help-Seeking and less Physical Venting than boys and boys being more reactive compared to girls. Perry and Dollar [35] found the exact same results for these ER strategies, aligning with the general literature [41]. However, Perry and Dollar [35] found additional sex differences for Distraction, Mindfulness, and Physical Help-Seeking. This may be due to different gender socialization patterns in our sample, since societal norms shape how boys and girls regulate their emotions [43,45]. However, this reasoning would not fully align with the idea of socio-psychological and cultural similarities between the US and Western European countries [127,128,129]. Additionally, contextual factors, such as family dynamics or social support [53,144], may have influenced strategy use, reducing sex differences. Furthermore, item wording or item interpretation could also have played a role because of the translation process. Lastly, it is possible that sex differences in these strategies were small and our sample lacked the statistical power to detect them. This would align with the idea that sex differences, even if significant, are small [46]. Future research should examine how social and methodological factors possibly influence sex differences in ER.

Unlike the original study [35], our study provides insight into how SES may influence ER patterns. Surprisingly, SES only influenced Emotional Reactivity, with a higher SES predicting more reactivity. This was not suspected, since previous studies reported strong associations between SES and adaptive and maladaptive strategies [56]. This inconsistency could stem from sample differences, such as a narrower SES range in our study. Cuartes et al. [52], for example, only found a significant difference in ER use between the lowest and highest SES quartiles. Moreover, these authors found that the influence of SES on ER differed for younger and older children. In addition, cultural factors might have influenced how SES impacts regulation. For instance, cultures that emphasize the collective good or have a strong social support system may lessen the influence of individual SES differences on the emotional development. Furthermore, other variables, like parental style, coregulation in general, or stress exposure, might have mediated the relationship between SES and ER [55,57]. Coregulation could potentially buffer for the negative effects of low SES on the ER of a child. Further research should explore these relationships using a diverse sample with a broader SES range to better understand the mechanisms linking SES to ER.

Overall, these findings suggest that age and sex are key predictors of some ER strategies, with older individuals showing more adaptive strategies such as Mindfulness and less reliance on maladaptive strategies such as Venting. Sex differences emerge particularly in Help-Seeking and Emotional Reactivity while SES appears to play a more limited role. While our study did not explicitly examine the impact of cultural aspects on ER strategy development, our findings emphasize the importance of cultural considerations in future research on ER strategy use itself but also on its relationship with demographics.

### 4.4. Limitations

While the dataset included around 300 preschool children, comparable to the original EERBQ study [35], a larger sample could have offered more nuanced insights into the CFA. In addition, although the model fit for all ER strategies was good, some factor loadings were lower than ideal. This may indicate that certain items did not strongly represent their intended strategy. Future research should examine whether the modification of items could improve the reliability of these strategies. Another possible methodological limitation is the lack of a criterion validity analysis in this study. Although the current study did not include a criterion validity analysis to provide information about how well the EERBQ-Dutch correlates with well-established measures of ER, Perry and Dollar [35] examined correlations between the EERBQ and the Emotion Regulation Checklist [119]) and the Strengths and Difficulties Questionnaire [145], allowing us to assume similar correlations.

Sample-wise, although all layers of SES were reached, our sample primarily consisted of middle-class SES participants, which may limit the generalizability of our findings, especially to the lower- and higher-SES population. Follow-up studies could provide a more diverse sample to improve the generalizability of findings.

While taking into account different demographic aspects, namely, sex, age, and SES, other environmental factors, such as parental emotion socialization and coregulation, stress exposure, peer influence, and cultural aspects, may contribute to ER differences but were not examined. Not only could these factors influence the strategies directly, but some might act as mediators. In the future, research could investigate additional contextual factors influencing the ER strategy use and employ longitudinal studies with multi-method designs. Despite these limitations, our study provides evidence for a reliable measure of ER strategies in positive- and negative-emotion-eliciting situations for preschoolers, and provides valuable insights into the role of age, sex, and SES in ER.

## 5. Conclusions

The current study supports the reliability and validity of the EERBQ-Dutch in assessing preschoolers’ ER strategy use, as evidenced by the excellent CFA model fit. This confirms the robustness of ER strategies and suggests that these are well-defined within our sample. Regarding reliability, most of the strategies demonstrated strong internal consistency, reinforcing the stability of the measures used. Furthermore, the strong test–retest reliability adds to the robustness and temporal stability of the ER strategies, providing further confidence in their use for future research and clinical purposes.

Additionally, our study offers valuable insights into the role of age, sex, and SES in ER. Overall, it contributes to the growing body of ER research by enhancing understanding of both measurement and demographic aspects, highlighting important directions for future studies.

## Figures and Tables

**Table 1:** ER strategies questioned in the EERBQ-Dutch.

ER Strategy	Definition
Mindfulness	Self-awareness of one’s own feelings (e.g., acknowledging feeling sad)
Avoidance	Withdrawing from, hiding, or avoiding the source of arousal (e.g., becoming withdrawn, quiet, or retreating to a personal space)
Distraction	The child’s ability to independently avert attention away from the source of distress to an activity or object less arousing (e.g., find another activity to do or toy to play with)
Verbal Help-Seeking	Eliciting caregiver assistance in dampening emotional arousal through verbal communication such as conversation or questioning (e.g., initiating a conversation with the caregiver)
Physical Help-Seeking	Eliciting caregiver assistance in dampening emotional arousal through physical contact such as hugs or holding (e.g., holding caregiver’s hand when feeling scared)
Self-Soothing	Physical mechanism that is independently generated to calm oneself such as sucking or rubbing (e.g., sucking thumb or cuddling a blanket)
Verbal Venting	Releasing arousal in a verbal way such as yelling, screaming, or crying (e.g., screaming because caregiver refuses to buy candy at a grocery store)
Physical Venting	Releasing arousal in a physical way such as running, jumping, kicking, or hitting (e.g., hitting another child)

Note. Definitions are in accordance with the original Early Emotion Regulation Behavior Questionnaire [35].

**Table 2:** Descriptives and internal reliability (Cronbach’s alpha) for the eight ER strategies and the Emotional Reactivity scale.

Strategy	*M* (*SD*)	Range	*α*
Mindfulness	4.8 (1.4)	1.1–7.0	0.92
Avoidance	2.2 (0.9)	1.0–4.7	0.79
Distraction	3.9 (1.1)	1.3–6.8	0.84
Verbal Help-Seeking	4.7 (1.3)	1.0–7.0	0.90
Physical Help-Seeking	5.1 (1.0)	2.0–7.0	0.81
Self-Soothing	2.1 (1.3)	1.0–7.0	0.94
Verbal Venting	2.6 (1.2)	1.0–6.3	0.88
Physical Venting	2.5 (0.9)	1.0–5.6	0.83
**Emotional Reactivity**	3.6 (0.9)	1.7–5.6	0.66

**Table 3:** Item–total correlation for each item of the eight ER strategies based on the 12 hypothetical situations and for each of the 6 questions of the Emotional Reactivity scale.

Strategy	S1	S2	S3	S4	S5	S6	S7	S8	S9	S10	S11	S12
Mindfulness	0.49	0.69	0.65	0.65	0.70	0.66	0.65	0.68	0.66	0.67	0.74	0.77
Avoidance	0.47	0.42	0.23	0.53	0.58	0.49	0.32	0.63	0.29	0.45	0.31	0.48
Distraction	0.47	0.42	0.56	0.57	0.60	0.42	0.34	0.60	0.41	0.51	0.54	0.58
Verbal Help-Seeking	0.57	0.55	0.61	0.61	0.68	0.66	0.60	0.67	0.50	0.72	0.74	0.67
Physical Help-Seeking	0.33	0.42	0.39	0.58	0.54	0.43	0.33	0.53	0.57	0.45	0.55	0.58
Self-Soothing	0.67	0.79	0.63	0.71	0.77	0.79	0.79	0.79	0.76	0.76	0.65	0.78
Verbal Venting	0.60	0.43	0.51	0.61	0.60	0.55	0.58	0.67	0.61	0.58	0.57	0.61
Physical Venting	0.65	0.44	0.29	0.60	0.68	0.36	0.44	0.69	0.59	0.43	0.25	0.59
**Emotional Reactivity**	**Question 1**		**Question 2**		**Question 3**		**Question 4**		**Question 5**		**Question 6**	
	0.52		0.58		0.49		0.51		0.18		0.13	

**Table 4:** Interclass correlation coefficients for each ER strategy and the emotional reactivity scale as indicators for test–retest reliability.

Emotion Regulation Strategy	ICC	95% CI	*p*
Mindfulness	0.888	[0.816, 0.931]	<0.001
Avoidance	0.760	[0.606, 0.853]	<0.001
Distraction	0.804	[0.678, 0.880]	<0.001
Verbal Help-Seeking	0.938	[0.899, 0.962]	<0.001
Physical Help-Seeking	0.825	[0.713, 0.893]	<0.001
Self-Soothing	0.923	[0.874, 0.953]	<0.001
Verbal Venting	0.881	[0.805, 0.927]	<0.001
Physical Venting	0.836	[0.731, 0.900]	<0.001
**Emotional Reactivity**	0.855	[0.763–0.912]	<0.001

**Table 5:** Multiple regression results (unstandardized coefficients (B values)) for demographic predictors (age, sex, and SES) of ER strategies and the Emotional Reactivity scale.

Variable	Mindfulness	Avoidance	Distraction	Verbal Help-Seeking	Physical Help-Seeking	Self-Soothing	Verbal Venting	Physical Venting	Emotional Reactivity
Constant	2.09 ***	1.81 ***	4.17 ***	1.55 ***	5.41 ***	1.73 ***	3.68 ***	3.48 ***	3.45 ***
Age	0.04 ***	0.00	0.00	0.04 ***	−0.01	−0.00	−0.02 ***	−0.01 ***	0.00
Sex	0.16	−0.15	0.02	0.30 *	0.11	0.06	−0.27	−0.43 ***	−0.28 *
SES	0.01	0.01	−0.01	0.01	−0.00	0.01	0.00	0.20	0.01 *
R^2^	0.16	0.02	0.01	0.25	0.01	0.00	0.06	0.11	0.04
Adj. R^2^	0.15	0.01	0.00	0.24	0.00	−0.01	0.05	0.10	0.03
F(df = 3)	16.80 ***	1.92	1.25	30.46 ***	1.00	0.30	6.30 ***	10.87 ***	3.46 *

* *p* < 0.050, *** *p* < 0.001

## Data Availability

Requests to access the dataset should be directed to Prof. Dr. Kurt Eggers due to ethical considerations.
